# Effects of self-perceived psychological stress on clinical symptoms, cortisol, and cortisol/ACTH ratio in patients with burning mouth syndrome

**DOI:** 10.1186/s12903-023-03235-0

**Published:** 2023-07-22

**Authors:** Yeon-Hee Lee, Chon Suk

**Affiliations:** 1 Department of Orofacial Pain and Oral Medicine, Kyung Hee University, Kyung Hee University Dental Hospital, #613 Hoegi-dong, Dongdaemun-gu, Seoul, 02447 South Korea; 2 Department of Endocrinology, Kyung Hee University, Kyung Hee University Medical Center, #613 Hoegi-dong, Dongdaemun-gu, Seoul, 02447 Korea

**Keywords:** Burning mouth syndrome, Stress, Salivary flow rate, Cortisol, ACTH, Cortisol/ACTH ratio

## Abstract

**Background:**

Psychological stress is a crucial parameter in defining the symptoms of burning mouth syndrome (BMS). We hypothesized that the level of psychological stress in patients with BMS would correlate with severity of clinical symptoms, cortisol levels, and cortisol/ adrenocorticotropic hormone (ACTH) ratio. We aimed to comprehensively investigate the influence of clinical and hematologic parameters on the hypothalamic–pituitary–adrenal axis, particularly concerning the presence or absence of self-perceived psychological stress in patients with BMS. In addition, we aimed to identify parameters predicting psychological stress in these patients.

**Methods:**

One hundred and forty-one patients with BMS (117 women, 82.98%; 56.21 ± 13.92 years) were divided into psychological stress (*n* = 68; 55 females, 56.39 ± 12.89 years) and non-psychological stress groups (*n* = 73; 62 females, 56.03 ± 14.90 years), and inter- and intra-group statistical analyses were conducted. Significant predictors of psychological stress in patients with BMS were investigated through multiple logistic regression analysis.

**Results:**

The prevalence of xerostomia was significantly higher (67.6% vs. 34.2%, *p* < 0.001), while unstimulated salivary flow rate was lower (0.66 ± 0.59 vs. 0.91 ± 0.53 mL/min, *p* < 0.01) in the psychological stress group than in the non-psychological stress group. SCL-90R subscale values for somatization, hostility, anxiety, and depression, as well as cortisol and ACTH levels and the cortisol/ACTH ratio, were also higher in the psychological stress group (all *p* < 0.05). Above-mean values for cortisol (AUC = 0.980, 95%CI: 0.959–1.000) and cortisol/ACTH (AUC = 0.779; 95%CI, 0.701–0.856) were excellent predictors of psychological stress, with cortisol (*r* = 0.831, *p* < 0.01) and cortisol/ACTH (*r* = 0.482, *p* < 0.01) demonstrating substantial correlations. Above-average values for cortisol (OR = 446.73) and cortisol/ACTH (OR = 6.159) significantly increased incidence of psychological stress in patients with BMS (all *p* < 0.001).

**Conclusions:**

Among patients with BMS, xerostomia, decreased salivary flow rate, increased cortisol levels, and cortisol/ACTH ratio were associated with psychological stress, highlighting the psycho-neuro-endocrinological features of this condition. Cortisol and cortisol/ACTH ratio were strong predictors of psychological stress in patients with BMS.

**Supplementary Information:**

The online version contains supplementary material available at 10.1186/s12903-023-03235-0.

## Background

Burning Mouth Syndrome (BMS) is a chronic medical condition characterized by burning sensation, dysesthesia, dysgeusia, and pain in the oral mucosa, with no clinically evident pathological changes [1]. Patients with BMS often experience burning, tingling, annoyance, tenderness, or numbness in the oral mucosa. The International Association for the Study of Pain defines BMS as a burning pain in the tongue or other oral mucosa with normal tissue and specific laboratory findings lasting at least 4–6 months [2]. The onset of pain is spontaneous and bilateral, with no identifiable triggers. BMS symptoms typically arise from the anterior two-thirds and dorsal and lateral margins of the tongue, the front of the hard palate, and the labial region [3]. The global prevalence of BMS in previous studies ranged 0.6–15%, primarily affecting middle-aged and older adults aged 38–78 years [4]. The incidence of BMS increases with age in both sexes, but is more common in postmenopausal women, with a reported female-to-male ratio ranging from 3:1–16:1 [5]. Despite decades of research, no biomarkers or clear diagnostic and predictive criteria have been established for BMS.

The etiology of BMS is multifactorial and ambiguous, with several associated local, systemic, and psychological factors being reported [6]. It was initially considered a psychogenic issue until neuropathic mechanisms explaining its symptoms were proposed [7]. Physically, these mechanisms include peripheral small-diameter fiber neuropathy of the oral mucosa, pathological changes involving the trigeminal system, and central pain resulting from hypofunction of dopaminergic neurons in the basal ganglia [8]. Recent studies have elucidated several physical and psychological factors significantly associated with signs and symptoms of BMS [9, 10]. Psychological distress can exacerbate symptoms in chronic pain conditions, including BMS.

Additionally, symptoms of BMS and xerostomia often coincide, with two-thirds of patients with BMS complaining of xerostomia [11, 12]. Patients with BMS had a significantly lower unstimulated salivary flow rate (UFR) than controls [13]. Several studies have shown clear alterations in the quality and quantity of saliva in these individuals [1, 14]. Reduced salivary secretion or changes in composition may be associated with exacerbation of BMS symptoms due to increased tongue or oral mucosa irritation. Salivary secretion is psychoneurologically regulated [15]; however, according to international criteria, BMS diagnosis does not consider changes in quality or quantity of saliva [16]. Further research is needed to determine significance of these associations.

Emotional stress, although often unavoidable, can distort and exacerbate symptoms in patients with BMS. Previous studies have used various tools, such as the Depression Anxiety Stress Scale, the Perceived Stress Questionnaire, and Lipp’s Stress Inventory, to assess psychiatric disturbances in these patients, with consistent results showing that they experience higher levels of emotional stress compared with controls [10, 12, 17]. BMS is characterized by complex clinical features and psychological behavior resulting from interactions between neurophysiological mechanisms and psychological factors. Further, it negatively impacts patients’ quality of life, causing anxiety, depression, somatization, and reduced socialization [1]. The Symptom Checklist-90-Revised (SCL-90R), a widely used psychological self-reported questionnaire, assesses 90 symptoms, including nine symptomatic dimensions: somatization, obsessive-compulsive disorder, interpersonal sensitivity, depression, anxiety, hostility, phobic anxiety, paranoid ideation, and psychoticism [18]. Notably, our previous study showed no significant difference in the values of each item of the SCL-90R subscale between patients with BMS with and without sleep problems [9]. However, the self-reported Beck’s depression index, state anxiety, and trait anxiety questionnaires demonstrated higher levels of depression and anxiety in patients with BMS compared with controls [19]. Mechanisms associated with BMS symptoms may be better understood by investigating the response of the hypothalamic–pituitary–adrenal (HPA) axis to psychological distress in patients.

Cortisol is a neuroendocrine hormone that responds to psychological stress [20, 21]. While a recent meta-analysis found higher cortisol levels in patients with BMS compared with controls [22], factors such as inflammation, pain, and psychological stress may also influence cortisol levels. Thus, additional research is needed to determine whether cortisol can serve as a reliable biomarker of stress in patients with BMS. Under stress conditions, the amygdala signals the hypothalamus to release corticotropin-releasing hormone, activating the HPA axis. This hormone then stimulates the release of adrenocorticotropic hormone (ACTH) from the anterior pituitary gland, triggering release of cortisol from the adrenal cortex [23]. In Cushing’s syndrome, high cortisol/ACTH ratio indicates elevated cortisol secretion from the adrenal glands; this has been proposed as a potential diagnostic biomarker for the disease [24, 25]. Increased cortisol levels contribute to effective stress management through mobilization of glucose and tissue substrates for fuel, suppression of non-vital organ systems, and inflammation control. Furthermore, stress-induced hormonal responses can exacerbate neuropathic pain by enhancing central sensitization [26]. Prolonged or exaggerated stress responses can lead to cortisol dysfunction, widespread inflammation, and pain [21]. A previous study reported significantly lower salivary cortisol levels following BMS treatment using low-level laser therapy [27]. However, no study has comprehensively examined the clinical characteristics of psychological stress, blood cortisol, and ACTH levels in patients with BMS.

Therefore, psychological stress is a crucial parameter in defining BMS symptoms, alongside physical changes such as peripheral and central nervous system neuropathies. In this study, we hypothesized that psychological stress levels in patients with BMS correlate with severity of clinical symptoms, cortisol levels, and cortisol/ACTH ratio. Additionally, we aimed to investigate the potential correlation between psychological stress and xerostomia occurrence. To test our hypothesis, we examined differences in clinical symptoms between patients with and without psychological stress, and investigated clinical and hematological HPA axis-related factors correlating with psychological stress. Finally, we investigated factors correlated with psychological stress using a visual analog scale (VAS) and assessed subjective severity of symptoms.

## Methods

### Participants

For this observational study, we recruited 141 patients (117 women; mean age, 56.21 ± 13.92 years) who presented to the Department of Orofacial Pain and Oral Medicine (Kyung Hee University Dental Hospital, Seoul, South Korea) between August 1, 2017 and August 31, 2022. A researcher (YHL) with more than 10 years of clinical experience in orofacial pain diagnosed patients with BMS based on burning sensation or dysesthesia in the oral cavity without discernible clinical abnormalities. Patients were split into two groups based on presence or absence of self-perceived psychological stress, determined using dichotomous yes-or-no questions, such as “Do you currently experience daily self-perceived psychological stress?” Inclusion criteria were determined according to the International Classification of Headache Disorders 3 (ICHD-3). The study included participants with: [1] superficial intraoral pain for > 3 months, [2] persistent (> 2 h/day) burning pain, [3] no visible clinical changes in oral mucosa [4], and symptoms not caused by another ICHD-3 diagnosis [16].

Before laboratory tests, participants were instructed to refrain from consuming caffeine or nicotine for at least 4 h and alcohol for at least 24 h, and to complete inventories for surveying duration, type, intensity, and areas of BMS symptoms. Scalar et al. classified BMS symptoms into primary (essential/idiopathic) and secondary symptoms [4]. Patients with local/systemic pathological conditions that could affect salivary flow and cause secondary BMS symptoms were excluded. Therefore, exclusion criteria included smoking, uncontrolled hyperlipidemia and/or diabetes, history of radiation therapy involving the head or neck area, history of psychiatric disorders or use of psychotropic drugs, history of immunosuppressant and/or cytotoxic medication use, and communication difficulties.

### Sample size

For sample size calculation, we used G*Power software (ver. 3.1.9.7; Heinrich-Heine-Universität Düsseldorf, Düsseldorf, Germany). The minimum number of participants for significant between-group comparisons was 63 patients per subgroup, with a significance level of 5%.

### Study design

All participants underwent physical examinations, laboratory screening tests, and psychiatric assessments using the SCL-90R. Xerostomia and sleep problems were identified using well-formed self-reported checklists with questions such as “Do you currently experience daily oral dryness?” and “Do you currently have sleep problems?” This study was designed following Strengthening the Reporting of Observational Studies in Epidemiology (STROBE) guidelines [28]. The research protocol complied with the Declaration of Helsinki and was approved by the Institutional Review Board of the University Hospital (KHD IRB no. 1709-4). All patients provided informed consent.

### Clinical evaluation

A BMS questionnaire was used to assess subjective discomfort, aggravating or alleviating factors, symptom duration, and symptom area. Because BMS is a representative chronic pain disease, patients with symptoms lasting > 3 months were targeted. Patients with symptoms lasting > 6 months were considered to have chronic BMS, and the ratio of patients with symptoms lasting for 3 vs. 6 months was determined based on presence or absence of psychological stress (Table 1). Severity of oral pain was determined using the visual analog scale (VAS) (with sores ranging from 0 to 10, the latter indicating the most severe imaginable pain) [29].

**Table 1:** Demographics and salivary flow rates in patients with BMS

	Non-psychological stress (***n***=73)	Psychological stress (***n***=68)	
	Mean ± SD or n (%)	Mean ± SD or n (%)	***p***-value
*Demographics*			
Male ^a^	11 (15.1%)	13 (19.1%)	0.655
Female ^a^	62 (84.9%)	55 (80.9%)	
Age (years) ^b^	56.03 ± 14.90	56.39 ± 12.89	0.875
*Clinical characteristics*			
VAS ^b^	4.82 ± 2.00	5.76 ± 1.89	0.005**
Symptom duration (months) ^b^	25.38 ± 53.37	43.13 ± 131.34	0.289
Chronic condition ^a^	35 (47.9%)	37 (54.4%)	0.501
Xerostomia ^a^	25 (34.2%)	46 (67.6%)	<0.001***
Sleep problem ^a^	31 (42.5%)	33 (48.5%)	0.290
*Salivary flow rate*			
UFR (mL/min) ^b^	0.91 ± 0.53	0.66 ± 0.59	0.008**
SFR (mL/min) ^b^	1.51 ± 0.77	1.26 ± 0.88	0.079

^a^: Results were obtained using the chi-square test. ^b^: b Results obtained using the Mann–Whitney U test. **: *p*<0.01. ***: *p*<0.001. Statistical significance was set at *p*<0.05. The results are shown in bold. VAS, visual analog scale; *UFR* Unstimulated salivary flow rate, *SFR* Stimulated salivary flow rate; *n* number, *SD* Standard deviation

Clinical evaluation included oral examinations, panoramic radiography, and blood sampling. A dichotomous questionnaire was used to obtain information on stressful psychological conditions, xerostomia, sleep problems, and systemic disease factors. A separate questionnaire was administered to exclude other systemic factors causing burning pain or abnormal oral sensations. Areas in the oral cavity with BMS symptoms and associated alleviating or exacerbating factors were determined using a well-formed dichotomous questionnaire, and a dataset was constructed and statistically processed.

Psychological status of the participants was investigated using the SCL-90R [9]. Patients responded to 90 questions on a five-point Likert scale ranging from 0 (not at all) to 4 (extremely), specifying how much each item had bothered them within the past 7 days. The scale measures symptom intensity and evaluates nine psychological symptom dimensions: somatization (SOM), obsessive-compulsiveness (O-C), interpersonal sensitivity (I-S), depression (DEP), anxiety (ANX), hostility (HOS), phobic anxiety (PHOB), paranoid ideation (PAR), and psychosis (PSY).

UFR was obtained by measuring saliva collected in 10 min from the spitting method while the patient was at rest. Stimulated salivary flow rate (SFR) was determined by measuring saliva collected while chewing gum for 5 min [30].

### Laboratory parameters

Blood sampling was conducted between 9:00 and 11:00 am to minimize variability due to circadian rhythms. The test included a complete blood count with differential leukocyte counts and various hematological variables. Levels of gonadal hormones, including stress markers, such as cortisol, adrenocorticotropic hormone (ACTH), the cortisol/ACTH ratio, and antidiuretic hormone (ADH), were measured. Thyroid function tests (triiodothyronine [T3], thyroxine [T4], and thyroid-stimulating hormone [TSH] tests) were performed. Additionally, levels of trace elements, including serum folate, ferritin, and vitamin B12, were also measured. The reference ranges for each variable were as follows: T3: 81–197 ng/dL, T4: 4.6–13 ng/dL, TSH: 0.3–4.0 µIU/mL (micro-international units per milliliter), vitamin B12: 160–970 pg/mL, folate: 1.5–16.9 ng/mL, ferritin: 10–168 ng/mL, ADH: < 6.7 pg/mL, cortisol (morning): 5–27 µg/dL, and ACTH: 10–60 pg/mL.

### Statistical methods

Data was analyzed using SPSS Statistics for Windows (version 26.0; IBM Corp., Armonk, NY). Continuous variables are presented as means ± standard deviations (SD), while categorical variables are presented as frequencies and percentages. Differences between groups were examined using the chi-square test for categorical variables, and the *t*-test and Mann–Whitney U test for continuous variables. Fisher’s exact and chi-square tests were used to determine equality of proportions. Spearman’s correlation and Cramer’s V analyses were performed to determine factors correlating with psychological stress and VAS scores.

To assess the performance of models at the classification threshold (above the mean value of each laboratory parameter), we plotted receiver operating characteristic (ROC) curves and calculated the corresponding area under the curve (AUC) values. As a rule of thumb, AUC values were interpreted as follows: AUC = 0.5 (no discrimination), 0.6 ≥ AUC > 0.5 (poor discrimination), 0.7 ≥ AUC > 0.6 (acceptable discrimination), 0.8 ≥ AUC > 0.7 (excellent discrimination), and AUC > 0.9 (outstanding discrimination) [31].

A multiple logistic regression analysis was performed to evaluate the risk of psychological stress in patients with BMS. Each parameter was converted into a dichotomous variable to identify significant relative risk factors for psychological stress. Clinical signs and symptoms, and psychological and laboratory parameters, were simultaneously considered to obtain the odds ratio (OR) for high likelihood of psychological stress (dependent variable). A two-tailed *p*-value of less than 0.05 was considered statistically significant for all analyses.

## Results

### Demographics and salivary flow rate of patients with BMS

Of 141 patients, 82.9% were women (*n* = 117; mean age, 59.95 ± 13.38 years), and 17.1% were men (*n* = 24; mean age, 52.58 ± 16.15 years). Patients were divided into two groups based on the presence or absence of self-perceived psychological stress. The non-psychological stress group comprised 73 patients (62 women [84.9%]; mean age, 56.03 ± 14.90 years), and the psychological stress group comprised 68 patients (55 women [80.9%]; mean age, 56.39 ± 12.89 years). The mean age of all patients was 56.21 ± 13.92 years, and there was no significant age difference between the groups. VAS scores were significantly higher in the psychological stress group than in the non-psychological stress group (5.76 ± 1.89 vs. 4.82 ± 2.00, *p* = 0.005). However, no difference in symptom duration was observed between the groups. Some patients (45.4%) reported sleep problems, with no difference in prevalence between the groups. Notably, the rate of xerostomia occurrence in the psychological stress group was significantly higher (48.5% vs. 42.5%, *p* < 0.001), and UFR was significantly lower in the psychological stress group than in the non-psychological stress group (0.66 ± 0.59 vs. 0.91 ± 0.53 mL/min, *p* = 0.008). However, no significant difference in SFR was observed between the two groups (Table 1).

### Signs and symptoms of BMS

Where patients reported sites of BMS symptoms, the dorsal surface of the tongue was the commonest area (82.3% of all patients) where a burning sensation was reported. BMS symptoms involving the entire tongue were more frequent in the psychological stress group than in the non-psychological stress group (5.5% vs. 17.6%, *p* = 0.032). Conversely, incidence of symptoms in the lips was higher in the non-psychological stress group (12.3% vs. 8.8%, *p* = 0.005). The commonest aggravating factor for BMS symptoms was irritating or spicy food (37.6%), while cold water was the commonest alleviating factor (25.5%). No significant difference in the frequency of factors was observed between the groups (Table 2).

**Table 2:** Comparison of clinical characteristics of patients with BMS according to the presence of psychological stress

	Non-psychological stress (*n*=73)	Psychological stress (*n*=68)	
	Mean ± SD or n (%)	Mean ± SD or n (%)	*p*-value
*Area (multiple choices allowed)*			
Tongue, dorsal surface	57 (75.3%)	59 (86.8%)	0.129
Tongue, lateral border	6 (8.2%)	11 (16.2%)	0.197
Tongue, entire	4 (5.5%)	12 (17.6%)	0.032*
Buccal mucosa	10 (13.7%)	17 (25.0%)	0.068
Gingiva	16 (21.9%)	10 (14.7%)	0.188
Lips	21 (28.8%)	7 (10.3%)	0.005**
Hard palate	9 (12.3%)	6 (8.8%)	0.346
*Aggravating factors (one choice allowed)*			
None	20 (27.4%)	20 (29.4%)	0.589
Emotional distress	16 (21.9%)	13 (19.1%)	
Irritating or spicy food	27 (36.9%)	26 (38.2%)	
Toothpaste or gargle solution	5 (6.9%)	4 (5.9%)	
Physical fatigue	5 (6.9%)	5 (7.4%)	
*Alleviating factors (one choice allowed)*			
None	35 (47.9%)	29 (42.6%)	0.248
Taking rest	14 (19.2%)	8 (11.8%)	
Cold water	15 (20.5%)	21 (30.9%)	
Taking a nap	4 (5.5%)	3 (4.4%)	
Focus on other things	4 (5.5%)	3 (4.4%)	
Use of chewing gum and candy	1 (1.4%)	4 (5.9%)	

Results were analyzed using the chi-square test. Statistical significance was set at *p* < 0.05. The results are shown in bold. *: *p* <0.05. **: *p*-value <0.01. Statistical significance was set at *p* < 0.05

### Comparison of SCL-90R profile between the patients

Compared with those without psychological stress, patients with psychological stress exhibited greater instability in seven of the nine SCL-90R parameters. The psychological stress group displayed significantly higher SOM, O-C, I-S, DEP, ANX, HOS, and PAR values than the non-psychological stress group (all *p* < 0.05). PHOB and PSY scores were not significantly different between the groups (Table 3).

**Table 3:** Comparison of psychological profile and laboratory parameters in patients with BMS

	Non-psychological stress (n=73)	Psychological stress (n=68)	
	Mean ± SD or n (%)	Mean ± SD or n (%)	*p*-value
*SCL-90R*			
SOM	45.71 ± 9.49	51.19 ± 13.09	0.006**
O-C	43.03 ± 8.42	46.28 ± 9.91	0.038*
I-S	42.07 ± 8.17	46.07 ± 10.43	0.013*
DEP	43.49 ± 10.32	48.66 ± 12.79	0.010*
ANX	44.75 ± 8.78	48.62 ± 10.89	0.023*
HOS	45.14 ± 10.08	51.96 ± 17.89	0.007**
PHOB	53.81 ± 59.15	56.24 ± 46.37	0.786
PAR	42.56 ± 6.72	46.07 ± 13.02	0.049*
PSY	43.86 ± 7.94	47.25 ± 14.16	0.086
*Laboratory parameters*			
T3 (ng/dL)	143.18 ± 23.09	169.93 ± 159.67	0.212
T4 (ng/dL)	6.85 ± 1.42	65.16 ± 236.98	0.161
TSH (μIU/mL)	2.10 ± 1.41	36.72 ± 183.45	0.156
Vitamin B12 (pg/mL)	727.12 ± 350.24	682.38 ± 293.69	0.458
Folate (ng/mL)	10.14 ± 6.12	43.48 ± 182.24	0.169
Ferritin (ng/mL)	66.63 ± 57.89	126.30 ± 228.57	0.058
ESR (mm/hr)	19.40 ± 36.71	21.49 ± 16.89	0.353
ADH (pg/mL)	3.89 ± 2.43	5.71 ± 4.20	0.007**
Cortisol (μg/dL)	6.39 ± 2.61	14.02 ± 4.04	<0.001***
ACTH (pg/mL)	35.56 ± 19.51	44.33 ± 22.05	0.016*
Cortisol/ACTH	0.23 ± 0.19	0.39 ± 0.25	<0.001***

The results were obtained using the Mann–Whitney U test. *: *p*<0.05. **: *p*<0.01. ***: *p*<0.001. Statistical significance was set at *p*<0.05. The results are shown in bold. *SCL-90R* Symptom checklist-90-revised, *SOM* Somatization, *O-C* Obsessive-compulsive, *I-S* Interpersonal sensitivity, *DEP* Depression, *ANX* Anxiety, *HOS* Hostility, *PHOB* Phobic anxiety, *PAR* Paranoid ideation, *PSY* Psychosis, *T3* Triiodothyronine, *T4* Thyroxine, TSH, Thyroid-Stimulating Hormone, *ESR* Eerythrocyte sedimentation rate, *ADH* Antidiuretic hormone, ACTH Adrenocorticotropic hormone, n number, *SD* Standard deviation.

### Comparison of laboratory parameters

**Fig. 1:**
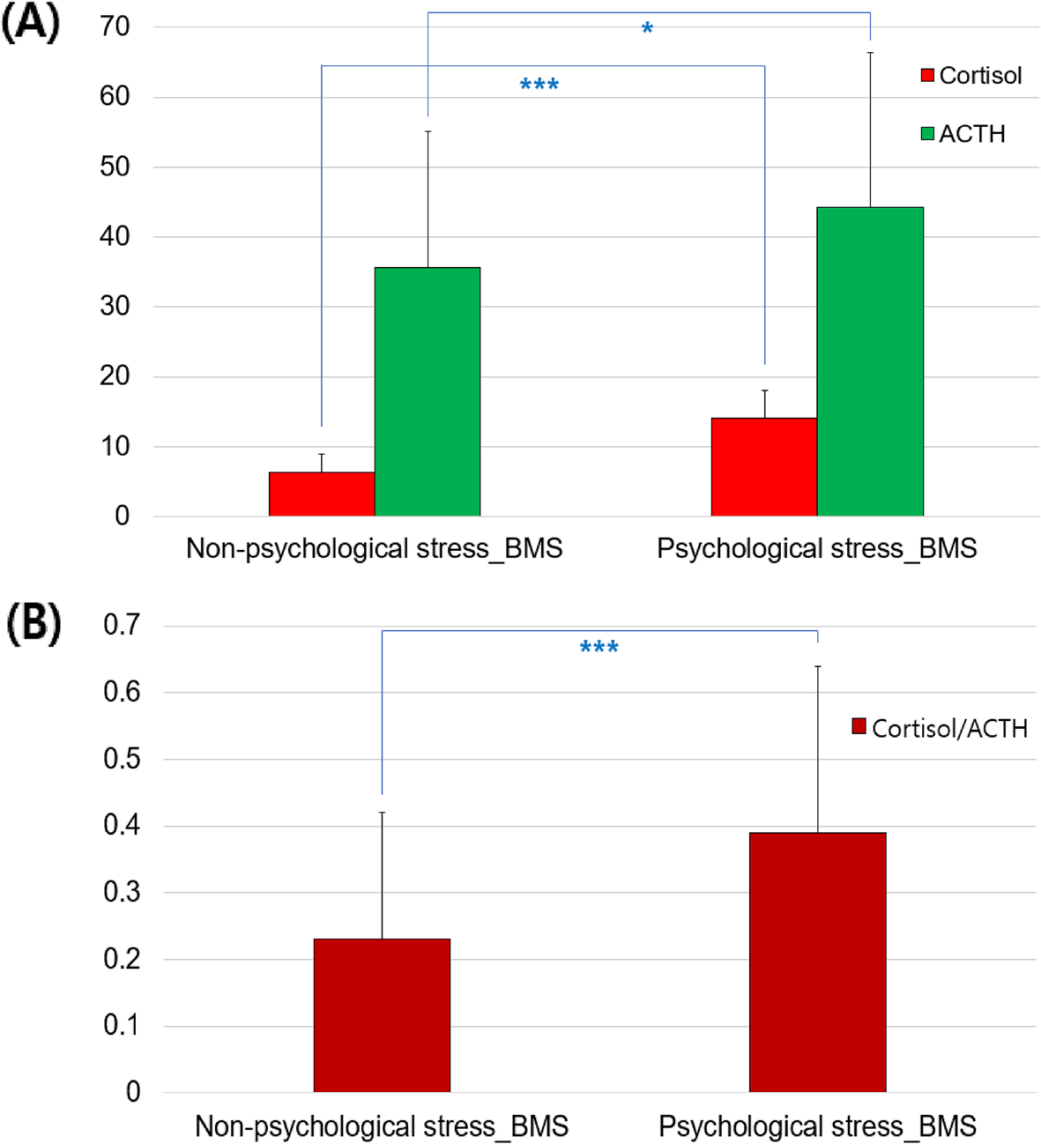
Comparison of cortisol, ACTH, and cortisol/ACTH ratio according to psychological stress. **A** cortisol and ACTH, and (**B**) cortisol/ACTH ratio. *: *p*-value < 0.05. ***: *p*-value < 0.001

Among laboratory parameters, ADH, cortisol, ACTH levels, and cortisol/ACTH ratio were higher in the psychological stress group than in the non-psychological stress group. Specifically, cortisol levels (6.39 ± 2.61 vs. 14.02 ± 4.04 µg/dL) and cortisol/DHEA ratios (0.23 ± 0.19 vs. 0.39 ± 0.25) varied significantly at *p* < 0.001 depending on the presence or absence of psychological stress (Fig. 1). No significant differences in levels of thyroid function markers (T3, T4, or TSH levels), or those of vitamin B12, folate, ferritin, and ESR, were observed between the groups (all p > 0.05). Although the average ESR value (21.49 mm/hr) was higher than the normal range (0–20 mm/hr) in the psychological stress group, average values of other laboratory parameters were within normal ranges (Table 3). The distribution of normal, low, and high values for hematological parameters according to normal ranges is shown in Supplementary Table 1.

### Cut-off values of cortisol, ACTH, and cortisol/ACTH ratio for psychological stress

**Fig. 2:**
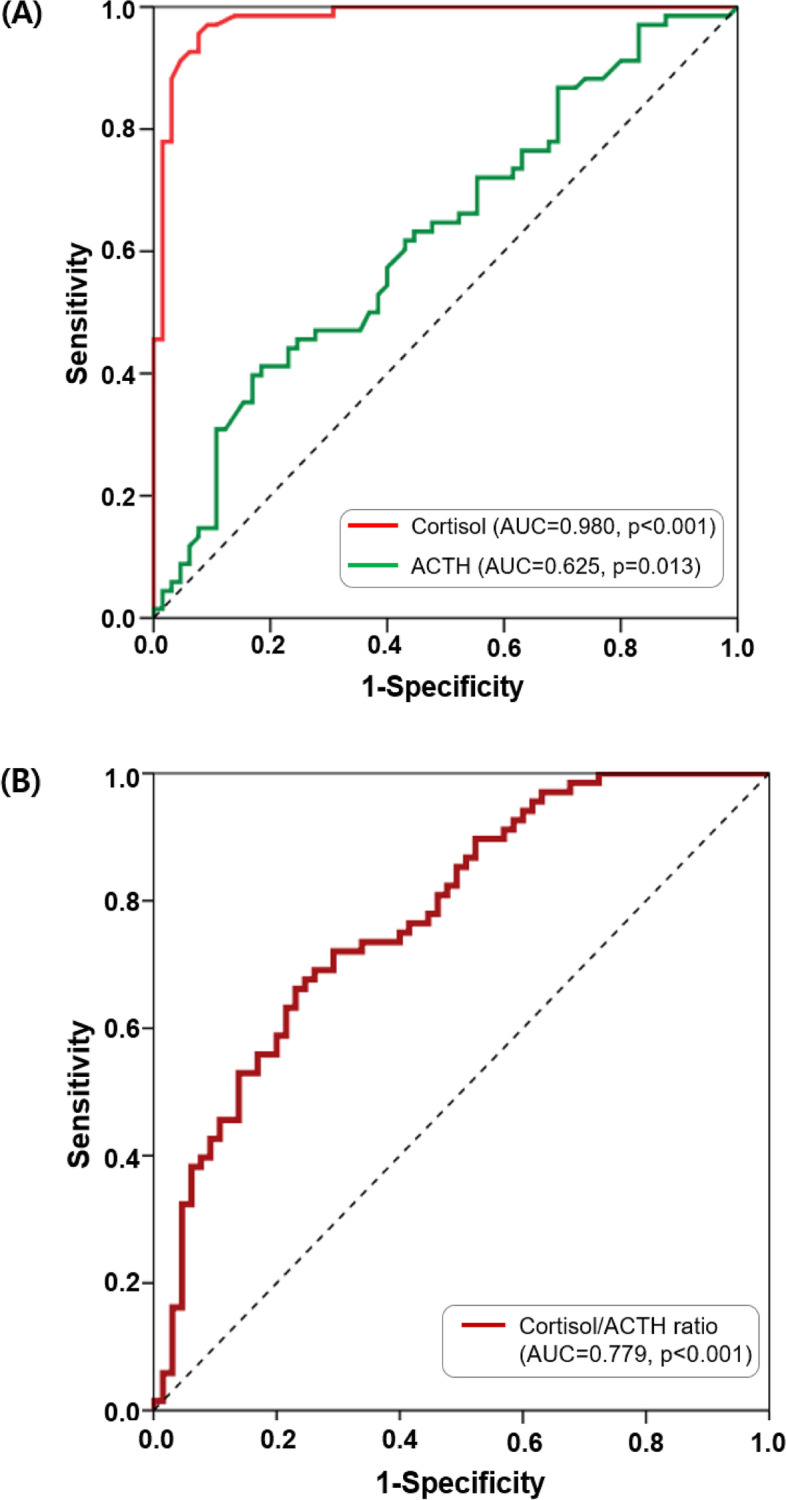
ROC curve and AUC of cortisol, ACTH, and cortisol/ACTH to predict psychological stress. **A** cortisol and ACTH, and (**B**) cortisol/ACTH ratio

Each laboratory parameter was coded ‘1’ when the average value was higher than the mean and ‘0’ when the value was lower. Cortisol (AUC = 0.980, 95% CI: 0.959, 1.000), and ACTH levels above the mean (AUC = 0.625, 95% CI: 0.530, 0.720), as well as a cortisol/DHEA ratio above the mean (AUC = 0.779, 95% CI: 0.701, 0.856) were found to be significant predictors of psychological stress in patients with BMS. Above-average cortisol values demonstrated outstanding discrimination in predicting psychological stress in patients with BMS, while the cortisol/ACTH ratio showed excellent discrimination (Fig. 2).

### Multivariate logistic regression analysis of factors influencing psychological stress among patients with BMS

We investigated factors influencing psychological stress among patients with BMS using multivariate logistic regression analysis. Two models were examined, one with cortisol and ACTH as independent variables and the other with cortisol/ACTH ratio as an independent variable. Above-average cortisol levels increased incidence of psychological stress in patients with BMS by 446.73 times (OR = 446.730, 95% CI: 53.765, 3711.871, p-value < 0.001). Above-average cortisol/ACTH levels increased incidence of psychological stress in patients with BMS by 6.159 times (OR = 6.159, 95% CI: 2.413, 15.721, p-value < 0.001) (Table 4).

**Table 4:** Multivariate logistic regression analysis of factors influencing psychological stress among patients with BMS

Model I for predicting psychological stress		95% CI			Model II for predicting psychological stress		95% CI		
(***n***=141)	OR	Lower	Upper	*p*-value	(***n***=141)	OR	Lower	Upper	*p*-value
VAS [ref.=under average value]	4.728	0.401	55.722	0.217	VAS [ref.=under average value]	0.606	0.134	2.748	0.516
UFR [ref.=under average value]	1.449	0.310	6.772	0.637	UFR [ref.=under average value]	1.419	0.577	3.487	0.446
Xerostomia [ref.=none]	0.413	0.031	5.522	0.504	Xerostomia [ref.=none]	2.277	0.440	11.793	0.327
BMS symptoms on the lips [ref.=none]	0.507	0.067	3.826	0.510	BMS symptoms on the lips [ref.=none]	0.811	0.289	2.276	0.690
BMS symptoms on the entire tongue [ref.=none]	0.916	0.114	7.382	0.934	BMS symptoms on the entire tongue [ref.=none]	1.430	0.408	5.013	0.576
SOM [ref.=under average value]	0.654	0.058	7.384	0.731	SOM [ref.=under average value]	2.071	0.517	8.299	0.304
O-C [ref.=under average value]	0.487	0.043	5.524	0.562	O-C [ref.=under average value]	0.581	0.151	2.239	0.430
I-S [ref.=under average value]	1.109	0.124	9.901	0.926	I-S [ref.=under average value]	1.950	0.515	7.375	0.325
DEP [ref.=under average value]	5.721	0.340	96.210	0.226	DEP [ref.=under average value]	2.421	0.647	9.059	0.189
ANX [ref.=under average value]	1.490	0.073	30.353	0.795	ANX [ref.=under average value]	0.522	0.107	2.545	0.421
HOS [ref.=under average value]	2.115	0.069	64.516	0.668	HOS [ref.=under average value]	3.513	0.451	27.358	0.230
PAR [ref.=under average value]	0.408	0.021	7.823	0.552	PAR [ref.=under average value]	0.217	0.037	1.278	0.091
**Cortisol [ref.=under average value]**	**446.730**	**53.765**	**3711.871**	<0.001***	**Cortisol/ACTH [ref.=under average value]**	**6.159**	**2.413**	**15.721**	<0.001***
ACTH [ref.=under average value]	2.472	0.473	12.934	0.284	constant	0.303			0.003
constant	0.045			0.000					

Results were obtained using a multivariate logistic regression analysis. *: *p*<0.05. ***: *p*<0.001. Statistical significance was set at p<0.05. The results are shown in bold. UFR, unstimulated salivary flow rate; *BMS* Burning mouth syndrome, *SOM* Somatization, *O-C* Obsessive-compulsive, *I-S* Interpersonal sensitivity, *DEP* Depression, *ANX* Anxiety, *HOS* Hostility, *PAR* Paranoid ideation, *ACTH* Adrenocorticotropic hormone, *ref* reference, *CI* Confidence interval. Model I: dependent variable=psychological stress; independent variables; cortisol and ACTH were treated as separate variables. Model II: dependent variable=psychological stress, independent variables, and cortisol/ACTH ratio.

### Factors correlated with psychological stress in patients with BMS

**Fig. 3:**
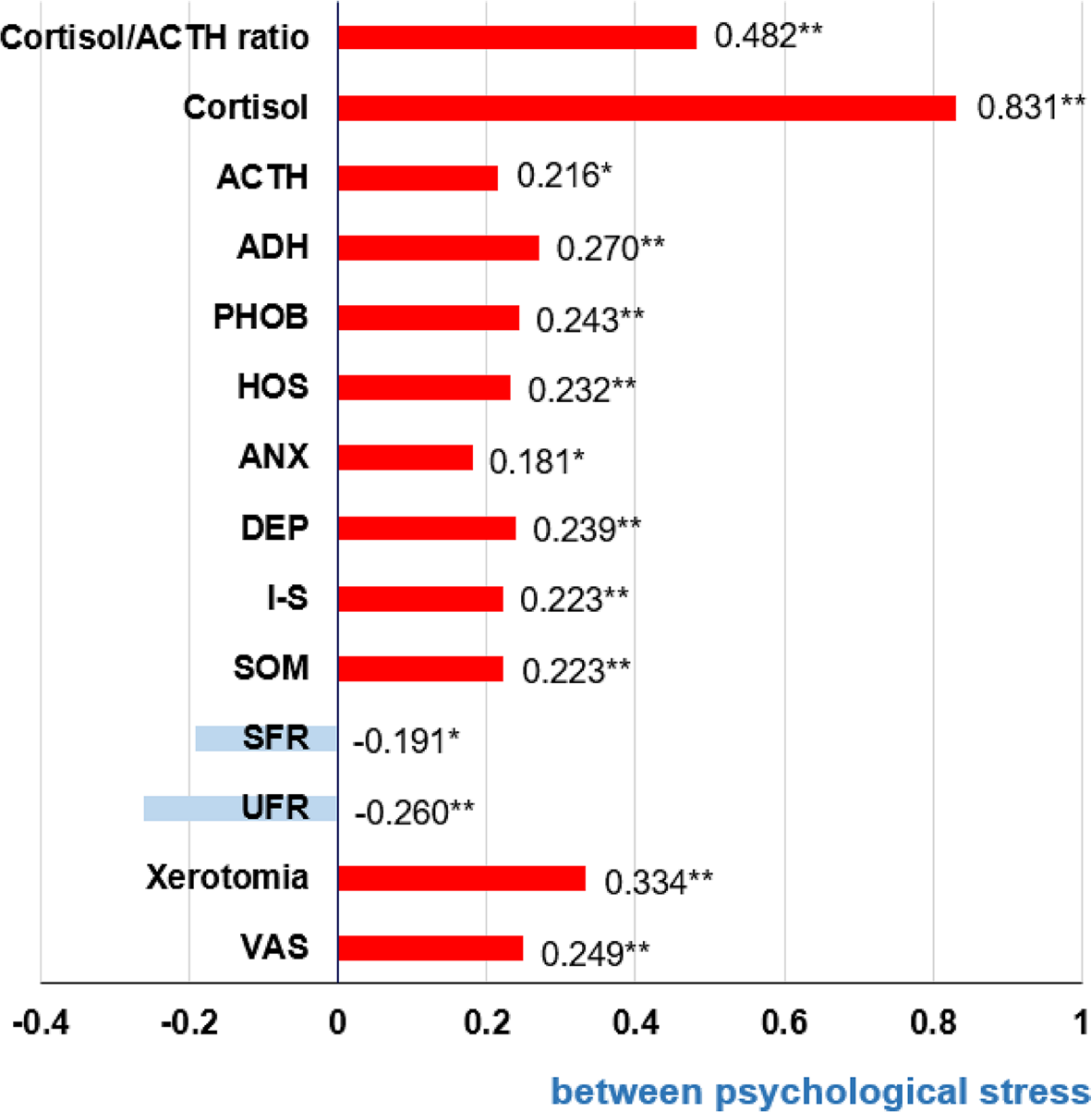
Correlation coefficient between psychological stress and other factors in patients with BMS. *: *p*-value < 0.05. **: *p*-value < 0.01

The correlation coefficients (r) for factors correlating with psychological stress were as follows: cortisol (*r* = 0.831), cortisol/ACTH ratio (*r* = 0.482), xerostomia (*r* = 0.334), ADH (*r* = 0.270), VAS (*r* = 0.249), PHOB (*r* = 0.243), DEP (*r* = 0.239), ANX (*r* = 0.232), SOM (*r* = 0.223), I-S (*r* = 0.223), ACTH (*r* = 0.216), and ANX (*r* = 0.181) (all *p* < 0.05). Salivary flow rate showed a significantly negative correlation with presence of psychological stress, with the UFR (*r*=–0.26, *p* < 0.01) having a stronger negative correlation with psychological stress than the SFR (r=–0.191, *p* < 0.05) (Fig. 3).

### Factors that increase VAS scores in patients with BMS

The presence of xerostomia was positively correlated with increased VAS scores in the non-psychological (*r* = 0.791, *p* < 0.01) and psychological stress groups (*r* = 0.684, *p* < 0.01). Meanwhile, increased VAS scores positively correlated with presence of sleep problems in both groups (non-psychological stress *r* = 0.474, *p* < 0.01; psychological stress *r* = 0.524, *p* < 0.01). In the non-psychological stress group, increased cortisol/ACTH ratio correlated significantly with increased VAS score (*r* = 0.261, *p* < 0.05). In the psychological stress group, symptom duration correlated positively with VAS score (*r* = 0.295, *p* < 0.05) (Table 5).

**Table 5:** Factors that increase VAS score in patients with BMS

Correlation with VAS	Demographics and Clinical characteristics				Psychological profile							Laboratory parameter
	Age^**a**^	Xerostomia^**b**^	Sleep problem^**b**^	Symptom duration^**a**^	SOM^**a**^	O-C^**a**^	I-S^**a**^	ANX^**a**^	HOS^**a**^	PAR^**a**^	PSY^**a**^	Cortisol/ACTH ratio
Non-psychological stress (n=73)												
r	.246^*^	.791^**^	.474^**^	.090	.298^*^	.286^*^	.279^*^	.177	.245^*^	.236^*^	.218	.261^*^
*p*-value	.036	.000	.000	.451	.011	.014	.017	.134	.037	.044	.064	.036
Psychological stress (n=68)												
r	.042	.684^**^	.524^**^	.295^*^	.239	.151	.187	.274^*^	.300^*^	.246^*^	.245^*^	.001
*p*-value	.731	.000	.000	.015	.050	.218	.128	.024	.013	.043	.044	.994

^a^Results were obtained using Spearman’s correlation analysis. ^b^Results were obtained using Cramer’s V analysis. *: *p*<0.05. **: *p*<0.01. Statistical significance was set at *p*<0.05. The results are shown in bold. VAS, visual analog scale; *SOM* Ssomatization, *O-C* Obsessive-compulsive, I-S Interpersonal sensitivity, *ANX* Anxiety, *HOS* Hostility, *PAR* Paranoid ideation, *PSY* Psychosis, *ACTH* Adrenocorticotropic hormone.

## Discussion

BMS is a unique and complex chronic medical condition requiring continued scientific research, and presenting with symptoms such as xerostomia, dysesthesia, dysgeusia, sleep problems, and psychological distress [9, 11, 32]. It is often associated with the absence of noticeable pathological changes, confusing clinicians and patients. In this study, the incidence of xerostomia was significantly higher, and that of UFR was significantly lower, in the psychological stress group than in the non-psychological stress group. This suggests that, in addition to subjective oral dryness, stress leads to objective salivation deterioration at rest in patients with BMS. SCL-90R results indicated significantly higher somatization, hostility, anxiety, and depression in the psychological stress group than in the non-psychological stress group. Among laboratory parameters, significantly higher levels of ADH, cortisol, and ACTH and a higher cortisol/ACTH ratio were observed in the psychological stress group compared with the non-psychological stress group. Notably, cortisol and cortisol/ACTH ratio strongly predicted psychological stress in patients with BMS. Cortisol showed the strongest correlation with psychological stress, while the cortisol/ACTH ratio also showed a positive correlation.

Cortisol levels performed outstandingly, while cortisol/ACTH ratio performed excellently, in predicting psychological stress in patients. Hormonal and salivary secretions are regulated by changes in the HPA axis, which should be considered when managing patients with BMS [33]. Cortisol, a hormone released by the adrenal cortex, regulates homeostasis during emotional and physical stress. Approximately 30 min after the onset of stress, cortisol levels peaked systemically and remained elevated for several hours [34]. Cortisol is a commonly used stress marker, but recent studies have suggested its potential value as a biomarker of BMS [22]. Cortisol levels have been found to reduce with improved BMS symptoms. Further, patients with BMS display higher salivary cortisol levels compared with controls [35].

Conversely, salivary cortisol levels and VAS scores were significantly lower after low-level laser therapy [27]. Although cortisol is a potential BMS biomarker, further well-designed studies are needed to evaluate its association with psychological stress and symptom severity. The pituitary gland secretes ACTH, which plays a major role in the body’s stress response. However, in this study, ACTH did not correlate with psychological stress in patients with BMS; only the cortisol/ACTH ratio showed a positive correlation. The cortisol/ACTH ratio is useful for diagnosing Cushing syndrome and primary hypoadrenocorticism [24, 36]. Further research is needed to determine the significance of cortisol and the cortisol/DHEA ratio as BMS biomarkers.

In this study, levels of trace elements, such as vitamin B12, ferritin, and folate, and hematological factors related to thyroid function, including T3, T4, and TSH, did not differ significantly in the presence or absence of psychological stress. However, decreased levels of these parameters have been associated with increased BMS symptoms, and vitamin B deficiency has been observed in patients with BMS [37]. Further, vitamin B and zinc supplements appear to significantly reduce BMS symptoms [38]. In addition, serum iron, vitamin B12, and folic acid levels were significantly lower in patients with BMS than in healthy controls [39]. The influence of T3, T4, and TSH levels on BMS symptoms has also been studied [40]. A recent systematic review examined the usefulness of 54 biomarkers, divided into five categories: [1] pain biomarkers (including estradiol, progesterone, DHEA, and substance P), [2] stress biomarkers (including cortisol and alpha-amylase), [3] inflammatory biomarkers, [4] trace elements, anions, and chemical compounds, and [5] others. Among these, stress biomarkers were the only reliable indicators [22]. Moreover, psychological stress appeared to enhance the role of cortisol as a predictor for BMS. Except for cortisol, average values of the other substances were within normal ranges and did not significantly contribute to predictions of psychological stress.

Xerostomia is a common comorbidity of BMS, being associated with neuropathy rather than glandular issues [41]. In this study, xerostomia and decreased UFR correlated positively with psychological stress in patients with BMS. The interplay between psychological stress, xerostomia, and decreased UFR is complex and may exacerbate BMS symptoms. BMS symptoms are likely transmitted throughout trigeminal innervation, as evidenced by the histopathological findings of nociceptive or peripheral nerves in symptomatic patients [4, 42]. Amenábar et al. also reported reduced UFR in patients with BMS compared with controls [35]. However, several reports have indicated that subjective xerostomia was more prevalent in patients with BMS, and that no significant difference in UFR or SFR was found between individuals with and without BMS [43]. Since subjective xerostomia may be present without reduced salivary flow rate, further research is needed to determine the relationship between psychological stress, salivary flow rate, and xerostomia in patients with BMS.

According to our results, BMS symptom persistence correlates positively with VAS scores. Physical health and emotional well-being are closely linked; hence, chronic pain is closely associated with the development of psychological problems [44], with significant changes observed in neuroendocrine response and brain function and/or structure [45]. Therefore, chronic BMS pain may be associated with higher VAS scores under stressful conditions. Notably, distribution of sleep problems did not differ between the groups and was not a significant predictor of psychological stress. Sleep problems are considered a significant exacerbating factor in disease progression from acute to chronic levels [46]. Poor sleep quality may be related to an aggravated burning sensation in the oral cavity in patients with BMS [47]. However, the relationship between stress, depression, and sleep disturbance in patients with BMS has not been fully determined [10]. Therefore, further studies are needed to comprehensively examine sleep problems, psychological stress, and symptom severity in patients with BMS, before forming a conclusion.

In the SCL-90R questionnaire, depression, anxiety, interpersonal sensitivity, somatization, hostility, and phobic ideation were correlated with psychological stress in patients with BMS. Moreover, increases in anxiety, hostility, paranoid ideation, and psychosis correlated positively with higher VAS scores in the psychological stress group. Depression and anxiety are two common psychological conditions that play an important role in BMS [48]. Compared with healthy controls, patients with BMS had higher levels of depression, anxiety, and hostility, and scored lower on quality of life and susceptibility to pain catastrophism metrics [49, 50]. These findings across multiple studies support that BMS has a poorly understood underlying pathophysiology, likely involving neuropathic and psychogenic pathways. Future systematic studies are needed to determine the mechanisms responsible for heightened pain intensity in psychologically vulnerable patients with BMS.

This study has several limitations, such as its single-center design, which may limit generalizability of the findings. Moreover, the absence of age- or sex-matched healthy controls may have affected the interpretation of results. In addition, the assessment of psychological stress was based on dichotomous questions, which may not have captured the complexity of stress in patients. Future studies should implement more in-depth and sophisticated methods for testing psychological stress. Despite these limitations, this study is the first to comprehensively investigate clinical factors, laboratory parameters, and psychological profiles of patients with BMS. The use of well-established reference values for laboratory parameters and SCL-90R subscales provides a useful basis for further comparison and interpretation of study results.

## Conclusions

Our study suggests that cortisol levels and cortisol/ACTH ratio are strong predictors of psychological stress in patients with BMS. When diagnosing and treating a patient with BMS, in addition to physical examination, clinicians should consider conducting cortisol and ACTH tests, as these hormones could elucidate the neuropsychological state of the patient. However, additional multi-center studies with larger sample sizes are required to confirm these conclusions.

## Supplementary Information

**Additional file 1.**

## Data Availability

The datasets used and/or analyzed in the current study are available from the corresponding author upon reasonable request.

## Abbreviations

ACTH: Adrenocorticotropic hormone
ADH: Antidiuretic hormone
AUC: Area under the curve
ANX: Anxiety
BMS: Burning mouth syndrome
DEP: Depression
HOS: Hostility
HPA: Hypothalamic–pituitary–adrenal
ICHD-3: International Classification of Headache Disorders 3
I-S: Interpersonal sensitivity
O-C: Obsessive-compulsiveness
PAR: Paranoid ideation
PHOB: Phobic anxiety
PSY: Psychosis
ROC: Receiver operating characteristic
SFR: Stimulated salivary flow rate
SOM: Somatization
STROBE: Strengthening the Reporting of Observational Studies in Epidemiology
T3: Triiodothyronine
T4: Thyroxine
TSH: Thyroid-stimulating hormone
UFR: Unstimulated salivary flow rate
VAS: Visual analog scale
